# The Relationship between Dietary Habits and Periodontal Pathogens in a Sample of Romanian Children and Adolescents: A Cross-Sectional Study

**DOI:** 10.3390/children10111779

**Published:** 2023-11-02

**Authors:** Georgiana Veronica Motoc, Raluca Iulia Juncar, Abel Emanuel Moca, Ovidiu Motoc, Rahela Tabita Moca, Ioan Andrei Țig, Luminița Ligia Vaida, Mihai Juncar

**Affiliations:** 1 Doctoral School of Biomedical Sciences, University of Oradea, 1 Universității Street, 410087 Oradea, Romania; veromotoc@yahoo.com (G.V.M.); rahelamoca@gmail.com (R.T.M.); 2 Department of Dentistry, Faculty of Medicine and Pharmacy, University of Oradea, 10 Piața 1 Decembrie Street, 410073 Oradea, Romania; abelmoca@yahoo.com (A.E.M.); ovidiumotoc3@gmail.com (O.M.); nelutig@yahoo.com (I.A.Ț.); ligia_vaida@yahoo.com (L.L.V.); mihaijuncar@gmail.com (M.J.)

**Keywords:** periodontal pathogens, dietary habits, Romania

## Abstract

The role of diet in shaping oral microbiota and its potential contribution to the development of periodontal pathogens cannot be understated. This study aimed to explore the correlation between dietary habits and the prevalence of 11 periodontal pathogens among children and adolescents in Oradea, Romania. The identification of these pathogens was performed using the micro-IDent test kit, capable of detecting 11 specific periodontal pathogens. Bacterial sampling was conducted from the crevicular fluid in the morning, prior to brushing, followed by the completion of a brief questionnaire by parents. The questionnaire captured various aspects of the children’s eating habits, including meal frequency, consumption of sweets, and hydration levels. The collected samples were dispatched to the laboratory for analysis, which provided insights into the abundance of microorganisms. The study encompassed 60 participants aged between 2 and 18 years, with the majority reported by their parents to have regulated meal timings, frequent sugar intake, and adequate hydration. The findings revealed significant associations between certain dietary factors and the presence of specific periodontal pathogens. Notably, the absence of breastfeeding was linked with the detection of *Tannerella forsythia* and *Campylobacter rectus*. Furthermore, frequent consumption of sweets corresponded with the presence of *Capnocytophaga* spp., which was particularly observed in individuals consuming sweets 2–3 times a day. Insufficient age-appropriate hydration showed an association with the prevalence of *T. forsythia*, *Peptostreptococcus micros*, and *Capnocytophaga* spp. In this sample, it became evident that eating habits and diet influenced the presence of several periodontal pathogens. The lack of breastfeeding was predominantly associated with positive results for *T. forsythia* and *C. rectus*, while inadequate hydration correlated more frequently with the presence of *T. forsythia* and *P. micros.* Moreover, frequent consumption of sweets was linked to the presence of *Capnocytophaga* spp.

## 1. Introduction

Gingivitis stands as the most prevalent form of periodontal disease diagnosed in children and adolescents [1]. If left untreated, it can progress to periodontitis, potentially leading to tooth loss [2]. The prevalence of gingivitis varies significantly among different populations, reported at rates ranging from 28.5% [3] to as high as 91.0% [4]. Commonly implicated factors in the onset of periodontal diseases among children and adolescents include dental plaque, dental calculus, and poor oral hygiene [3]. Moreover, factors such as diabetes, lower family education levels, and socioeconomic status are associated with a higher risk of developing periodontal diseases [5].

While dental bacterial plaque plays a crucial role in the initiation of periodontal diseases [5], it also serves a defensive function against invading microbes in the oral environment [6]. Constituting part of the complex oral microbiome, dental plaque comprises a wide array of viruses, protozoa, fungi, and bacteria [7]. This intricate mix includes six major phyla, accounting for 96% of the oral cavity’s microbiome taxa—*Firmicutes*, *Bacteroidetes*, *Proteobacteria*, *Actinobacteria*, *Spirochaetes*, and *Fusobacteria*—that are typically commensal bacteria under normal conditions [7]. The disruption in the balance of this oral microbiome, known as dysbiosis, leads to the proliferation of periodontal pathogenic microorganisms like *Aggregatibacter actinomycetemcomitans*, *Porphyromonas gingivalis*, and *Tannerella forsythia*, precipitating periodontal disease [8,9]. Various commercial kits are available to accurately identify these periodontal pathogens [10].

Nutrition significantly influences the occurrence of oral dysbiosis and the development of pathogenic bacteria [11]. The consumption of foods that are high in added sugars heightens the risk of dysbiosis and subsequent periodontal disease, whereas the intake of whole grains and fruits decreases this risk [12]. A high sugar consumption has been associated not only with a higher incidence of periodontal disease among the pediatric population [13] but also with increased rates of dental caries [14], as well as systemic conditions like obesity, diabetes, and cardiovascular diseases [15]. Adequate hydration is crucial for maintaining body homeostasis [16], and individuals with higher water intake exhibit a reduced risk of developing periodontal disease and tooth decay [16].

Periodontal disease is multifactorial in its origins [17], but the role of nutrition and eating habits, particularly in children and adolescents, remains understudied. Few studies have concentrated on identifying periodontal pathogens among the pediatric population, specifically on investigating the role of nutrition in the development of periodontal pathogenic bacteria [18]. Geographical variations have been observed in the prevalence of gingivitis or periodontal disease among children and adolescents [3,4,19], as well as in the preferred composition of the oral microbiome [20]. Given these considerations, exploring the relationship between diet, eating habits, and various periodontal pathogens in children and adolescents from Oradea, Romania, holds vital importance and contributes valuable insights into periodontal pathology.

This study aims to investigate the association between diet, eating habits, and the prevalence of 11 periodontal pathogens in a sample of children and adolescents from Oradea, Romania.

## 2. Materials and Methods

### 2.1. Ethical Considerations

The research procedures adhered to the principles outlined in the Declaration of Helsinki, as per its guidelines established in 2008 and its most recent amendment in 2013. In compliance with these ethical standards, prior consent from the legal guardians or parents of children and adolescents under 18 years of age was obtained for their participation and the collection of crevicular fluid samples. Participation in the study incurred no costs for the participants and was entirely voluntary. Furthermore, the study received approval from the Research Ethics Committee of the Faculty of Medicine and Pharmacy at the University of Oradea (IRB No. CEFMF/10 dated 30 May 2022).

### 2.2. Study Design and Participants

The study, conducted on children and adolescents under 18 years of age in Oradea, Romania, was designed as a cross-sectional study. Crevicular fluid samples were collected between October 2021 and April 2022 at a private dental office in Oradea, Romania.

The study employed specific inclusion and exclusion criteria to ensure the participants met certain conditions. Inclusion criteria involved participants who were clinically healthy, free of systemic disorders, not receiving ongoing treatment for acute or chronic illnesses, residents of Romania, and whose parents provided consent for their involvement in the study.

Exclusion criteria were applied to eliminate participants based on specific circumstances. These included cases where the questionnaire was inaccurately or incompletely filled out, instances where the bacterial sample was compromised by medical staff, individuals undergoing orthodontic treatment, and teenagers who admitted to daily smoking habits.

Initially, the study included 71 children and teenagers. However, 11 were subsequently excluded due to various reasons: 4 had inaccurately or incompletely filled out questionnaires, 2 had compromised bacterial samples, 4 were undergoing orthodontic treatment, and 1 teenager admitted to daily smoking.

The participants were grouped into three distinct age categories: 0–5 years, 6–12 years, and 13–18 years. This categorization was chosen to encompass critical developmental periods in the lives of children and adolescents, namely preschool, school-age, and adolescence [21].

### 2.3. Questionnaire

Before the collection of crevicular fluid samples, parents were provided with a paper questionnaire in Romanian. The questionnaire, though not formally validated, was designed to gather pertinent information from the parents or legal guardians. It was filled out in the waiting room, and any uncertainties regarding the questions were addressed by the attending dentist.

The questionnaire encompassed sections for recording patient details such as age, gender, and specific information related to the duration of natural breastfeeding (no breastfeeding, breastfeeding up to 3 months, breastfeeding up to 6 months, breastfeeding up to 12 months, breastfeeding up to 18 months, and breastfeeding for more than 18 months).

Three questions concentrated on the current dietary habits of the children and teenagers, where responses were recorded as either “Yes” or “No.” These questions inquired whether the children adhered to fixed meal times, had a frequent intake of sweets (at least 2 times a day), and followed age-appropriate hydration guidelines (2–8 years: 1200 mL per day, 9–13 years: 1700 mL per day, 14–18 years: 1900 mL per day) [22].

The last two questions aimed to assess the frequency of daily meals (4 meals/day, 5 meals/day, more than 5 meals/day) and the frequency of sweet consumption per day (1/day, 2–3/day, more than 3/day). Parents selected one of the three available options for each question.

These responses were critical in gathering data regarding the dietary and hydration habits of the children and teenagers involved in the study.

### 2.4. Periodontal Pathogens

The investigation of periodontal pathogens utilized the micro-IDent test kit from Hain Lifescience, GmbH in Nehren, Germany. This kit, based on the polymerase chain reaction (PCR) technique, is capable of identifying 11 specific periodontal bacterial markers: *A. actinomycetemcomitans*, *P. gingivalis*, *Prevotella intermedia*, *T. forsythia*, *Treponema denticola*, *P. micros*, *Fusobacterium nucleatum/periodonticum*, *Eikenella corrodens*, *Campylobacter rectus*, *Eubacterium nodatum*, and *Capnocytophaga* spp. [23].

The collection of gingival crevicular fluid samples was conducted in the morning, prior to brushing, by a single operator, a Ph.D. student (G.V.M.). In order to ensure consistency and accuracy throughout the process, the operator underwent comprehensive training and procedural standardization in sample collection as part of the operator calibration process. This training was designed to familiarize the operator with the specific techniques and protocols required for the utilization of the Micro-IDent test kit from Hain Lifescience, GmbH, in Nehren, Germany. The operator was trained in the standardized collection of gingival crevicular fluid samples using the test kit’s paper sticks, following the manufacturer’s guidelines precisely. The proper method of sample collection, duration of placement in the sulcus, and subsequent transfer into the provided tubes was emphasized during this training.

The test kit’s paper sticks were used for sample collection according to the manufacturer’s guidelines. The sticks were placed in the sulcus for 10 s, then removed and inserted into the provided transfer tubes, along with a sheet containing the child’s information. These samples were then delivered to a laboratory, where they were kept stable for 7 days at 2 to 8 °C.

In the laboratory, the 1.5 mL vials containing the samples had a Chelex solution with a 5% concentration (150 mL) added. Following centrifugation and vortexing, the tubes were heated to 95 °C. A 5 mL volume from the supernatant solution was utilized for the subsequent PCR test. The PCR process involved amplification in a 50 μL reaction volume, comprising 5.0 μL of template DNA and a reaction mixture consisting of 35 μL of a primer-nucleotide-PNM (Micro-IDent) mixture, 5.0 μL of a 10× PCR buffer, 5.0 μL of 2.5 mM MgCl_2_, and 0.2 U Taq enzyme for the hot start provided by Qiagen, GmbH in Hilden, Germany. The GTQ-Cycler 96 thermal cycler from HAIN Lifescience was used for the PCR cycling.

Subsequently, reverse hybridization was carried out according to the Micro-IDent test instructions. Biotinylated amplicons were denatured and incubated at 45 °C with a hybridization buffer in a Twincubator, housing two control lines and six species-specific probes for Micro-IDent [24]. After nonspecific DNA binding was removed via a precise washing process, samples were cleaned, and alkaline phosphatase substrate was added. This step allowed for the observation of the hybridization results by adding alkaline phosphatase conjugated to streptavidin [25].

The laboratory provided the results containing the intensity of the microorganisms to the dental office, categorized as negative, weak positive, positive, or intense positive. A negative result suggested the absence of the targeted microorganism in the sample. A weak positive result indicated a low or borderline presence of the microorganism. A positive result signified a definite presence of the microorganism, and an intense positive result indicated a strong presence of the microorganism.

### 2.5. Statistical Analysis

Data organization involved Microsoft Office Excel 2013 (Microsoft, Redmond, DC, USA), and subsequent analysis was executed using IBM SPSS Statistics 26 (IBM, Chicago, IL, USA). Visual representations were created utilizing Microsoft Office Word 2013 (Microsoft, Redmond, DC, USA). Our study encompassed an examination of variables categorized into three distinct domains: demographic characteristics, dietary factors, and a classification system for identifying the scrutinized microorganisms.

All variables scrutinized in this study were characterized as nominal qualitative variables. Their distribution was ascertained by assessing discrete subgroups, measured in terms of absolute frequencies and relative frequencies, typically expressed as percentages. For instance, we considered four subgroups corresponding to the growth measurement of each microorganism (negative, weak positive, positive, intense positive). Additionally, two subgroups denoted the presence or absence of specific dietary factors, such as scheduled feeding patterns, daily sweet consumption, and adequate hydration. Six subgroups characterized the duration of breastfeeding, while three subgroups were created to encapsulate the frequency of daily meals or consumption of sweets.

Given the exclusive focus on qualitative variables, we abstained from conducting assessments related to quantitative variables, including but not limited to QQ plots, normality tests, and kurtosis/skewness evaluations, as there was a conspicuous absence of quantitative variables within our analytical framework. Consequently, the distribution of qualitative variables was determined in accordance with the previously described approach, primarily utilizing absolute or relative frequencies.

To evaluate discrepancies in frequency across subgroups, Fisher’s Exact Test was employed as our primary statistical tool. This choice was deliberate, as it accommodated scenarios featuring subgroups with zero observations, ensuring a precise calculation of probability values (*p*-values). This rendered it suitable for datasets of varied sizes. In contrast, the Pearson Chi-Square test, which relies on approximations, was unsuitable in specific instances.

Subsequently, when *p*-values fell below the predetermined alpha threshold for significance, usually set at 0.05, further analysis incorporated Z-tests with Bonferroni correction. These supplementary tests were instrumental in pinpointing observed frequency disparities among subgroups, especially when dealing with contingency tables exceeding the dimensions of 2 × 2.

Regarding sample size, formal calculations were not executed prior to data collection. Instead, a convenience sample comprising 50 patients was deemed sufficient to address the study’s objectives. In several instances, significant distinctions between subgroups were detected, thereby affirming the adequacy of the chosen sample size. Expanding the sample size was expected to yield results consistent with our findings.

In the preliminary stages, we considered univariable and multivariable multinomial logistic regression models to discern significant factors governing microorganism growth. These models were deemed invalid owing to the limited sample size and the prevalence of cells with zero observations. Consequently, we recategorized the growth of analyzed microorganisms as binary variables, thereby initiating a comprehensive reanalysis spanning descriptive statistics, contingency tables, and measures of association. Finally, univariable and multivariable binary logistic regression models were employed. Rigorous assessments of goodness-of-fit and significance were conducted to validate the robustness of these models.

## 3. Results

The final sample comprised 60 children and adolescents, with 23 boys (38.3%) and 37 girls (61.7%). These participants ranged in age from 2 to 18 years, distributed across three age groups: 2–5 years (n = 16, 26.7%), 6–12 years (n = 36, 60%), and 13–18 years (n = 8, 13.3%).

The duration of breastfeeding was also recorded. It was observed that 12 children (20.0%) were never breastfed naturally, 6 children (10.0%) were breastfed naturally for 3 months, 12 children (23.3%) were breastfed naturally for 6 months, 7 children (11.7%) were breastfed naturally for 12 months, 11 children (18.3%) were breastfed naturally for 18 months, and 10 children (16.7%) were breastfed naturally for more than 18 months.

**Figure 1:**
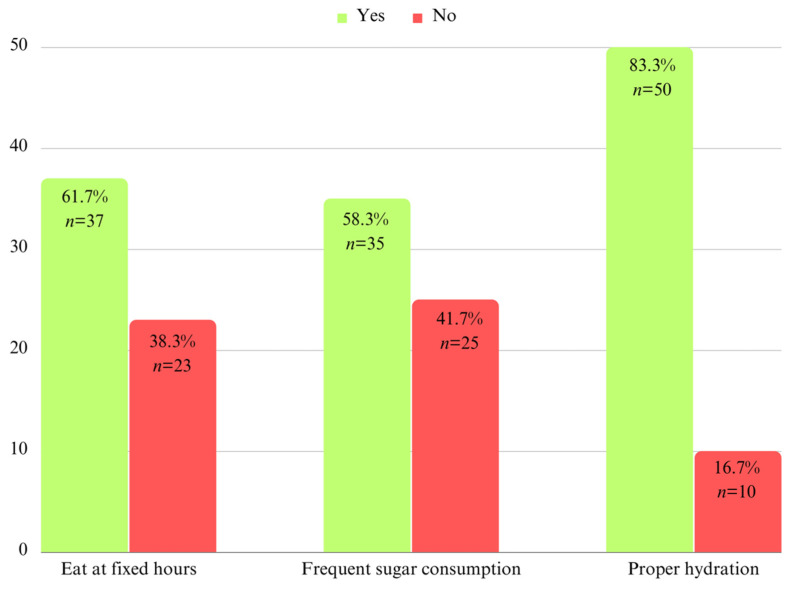
Eating habits.

**Figure 2:**
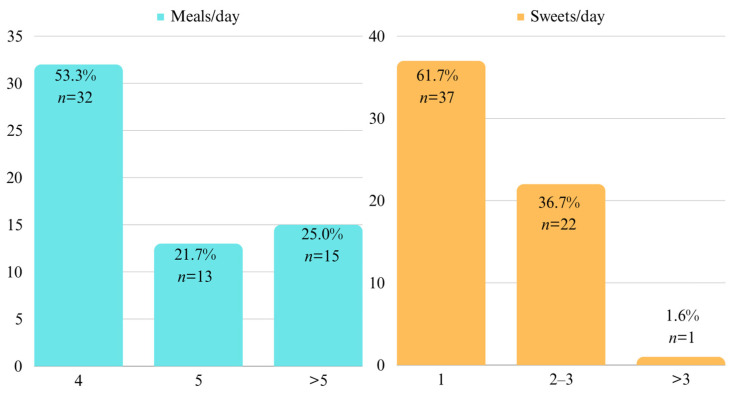
Frequency of food and sugar intake in a day.

The data in Figure 1 and Figure 2 show the eating habits of the children included in the study.

**Figure 3:**
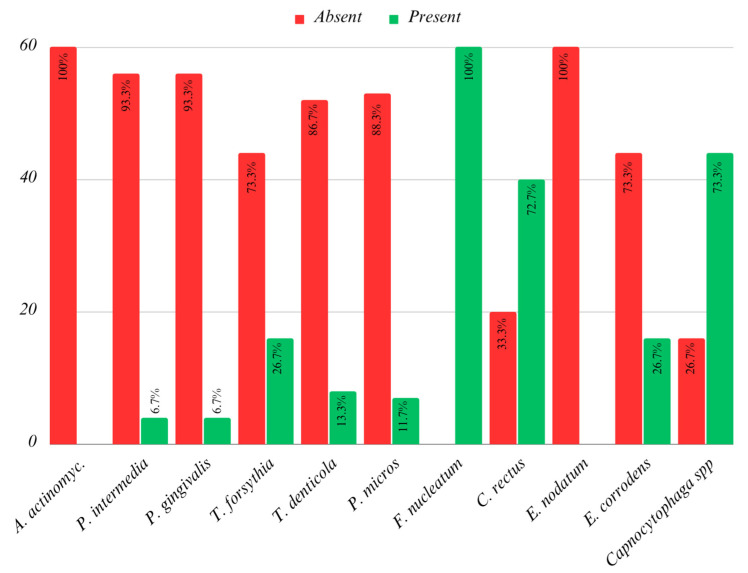
Identified periodontal pathogens.

The data presented in Figure 3 demonstrate the presence of various microorganisms identified in the collected samples, irrespective of the observed intensity (whether weak positive, positive, or intense positive). Below is a summary of the findings:

- *A. actinomycetemcomitans* was absent in 100% of cases.
- *P. intermedia* was found in 6.7% of cases.
- *P. gingivalis* was detected in 6.6% of cases.
- *T. forsythia* was present in 26.7% of cases.
- *F. nucleatum/periodonticum* was found in 100% of cases.
- *E. corrodens* was present in 26.7% of cases.

The data presented in Table 1 reveal the distribution of patients concerning the duration of breastfeeding and the intensity of the identified microorganisms. The statistical analysis, particularly utilizing Fisher’s test, was employed to assess the significance of differences between the groups. A breakdown of the findings and their implications is as follows: For the microorganisms *P. intermedia* (*p* = 0.494), *P. gingivalis* (*p* = 0.272), *T. denticola* (*p* = 0.219), *P. micros* (*p* = 0.071), and *Capnocytophaga* spp. (*p* = 0.087), the differences between the groups were not statistically significant according to Fisher’s test. This implies that, within the studied group, the intensity of these microorganisms did not significantly vary in relation to the duration of breastfeeding. However, for *T. forsythia* (*p* = 0.019), *F. nucleatum* (*p* = 0.002), *C. rectus* (*p* = 0.002), and *E. corrodens* (*p* = 0.013), the differences between the groups were statistically significant. Further analysis through Z tests with Bonferroni correction showed the following associations:

- *Tannerella forsythia*: A lack of breastfeeding was significantly more frequently associated with positive results, indicating its presence in the collected samples.
- *Fusobacterium nucleatum*: Breastfeeding durations of up to 3 months and up to 12 months were significantly more frequently associated with results indicating its presence in the collected samples.
- *Campylobacter rectus*: A lack of breastfeeding was significantly more frequently associated with results indicating the presence of this microorganism in the subgingival samples. Simultaneously, a duration of breastfeeding of up to 6 months was significantly more frequently associated with results indicating its absence from the collected samples.
- *Eikenella corrodens*: A lack of breastfeeding was significantly more frequently associated with its presence in the collected samples. Conversely, a duration of breastfeeding of up to 18 months was significantly more frequently associated with its absence from the collected samples.

**Table 1:** Distribution according to the duration of breastfeeding and identified microorganisms.

	*P. intermedia*	*P. gingivalis*	*T. forsythia*	*T. denticola*	*P. micros*	*F. nucleatum*	*C. rectus*	*E. corrodens*	*Capnocytophaga* spp.
Duration	Negative								
No breastf.	10 (17.9%)	12 (21.4%)	6 (13.6%)	8 (15.4%)	9 (17.0%)	0 (0.0%)	0 (0.0%)	5 (11.4%)	4 (25.0%)
3 months	6 (10.7%)	6 (10.7%)	6 (13.6%)	6 (11.5%)	6 (11.3%)	0 (0.0%)	2 (10.0%)	6 (13.6%)	0 (0.0%)
6 months	12 (21.4%)	10 (17.9%)	8 (18.2%)	12 (23.1%)	10 (18.9%)	0 (0.0%)	8 (40.0%)	10 (22.7%)	6 (37.5%)
12 months	7 (12.5%)	7 (12.5%)	7 (15.9%)	7 (13.5%)	7 (13.2%)	0 (0.0%)	2 (10.0%)	4 (9.1%)	2 (12.5%)
18 months	11 (19.6%)	11 (19.6%)	7 (15.9%)	9 (17.3%)	11 (20.8%)	0 (0.0%)	6 (30.0%)	11 (25.0%)	4 (25.0%)
>18 months	10 (17.9%)	10 (17.9%)	10 (22.7%)	10 (19.2%)	10 (18.9%)	0 (0.0%)	2 (10.0%)	8 (18.2%)	0 (0.0%)
	Weak positive								
No breastf.	0 (0.0%)	0 (0.0%)	2 (25.0%)	4 (50.0%)	1 (20.0%)	7 (38.9%)	7 (26.9%)	7 (43.8%)	3 (12.5%)
3 months	0 (0.0%)	0 (0.0%)	0 (0.0%)	0 (0.0%)	0 (0.0%)	0 (0.0%)	4 (15.4%)	0 (0.0%)	6 (25.0%)
6 months	0 (0.0%)	2 (100%)	2 (25.0%)	2 (25.0%)	4 (80.0%)	2 (11.1%)	2 (7.7%)	4 (25.0%)	5 (20.8%)
12 months	0 (0.0%)	0 (0.0%)	0 (0.0%)	0 (0.0%)	0 (0.0%)	3 (16.7%)	2 (7.7%)	3 (18.8%)	1 (4.2%)
18 months	0 (0.0%)	0 (0.0%)	4 (50.0%)	2 (25.0%)	0 (0.0%)	5 (27.8%)	3 (11.5%)	0 (0.0%)	4 (16.7%)
>18 months	0 (0.0%)	0 (0.0%)	0 (0.0%)	0 (0.0%)	0 (0.0%)	1 (5.6%)	8 (30.8%)	2 (12.5%)	5 (20.8%)
	Positive								
No breastf.	2 (50.0%)	0 (0.0%)	4 (50.0%)	0 (0.0%)	2 (100%)	5 (13.2%)	5 (35.7%)	0 (0.0%)	3 (18.8%)
3 months	0 (0.0%)	0 (0.0%)	0 (0.0%)	0 (0.0%)	0 (0.0%)	4 (10.5%)	0 (0.0%)	0 (0.0%)	0 (0.0%)
6 months	2 (50.0%)	2 (100%)	4 (50.0%)	0 (0.0%)	0 (0.0%)	12 (31.6%)	4 (28.6%)	0 (0.0%)	3 (18.8%)
12 months	0 (0.0%)	0 (0.0%)	0 (0.0%)	0 (0.0%)	0 (0.0%)	2 (5.3%)	3 (21.4%)	0 (0.0%)	3 (18.8%)
18 months	0 (0.0%)	0 (0.0%)	0 (0.0%)	0 (0.0%)	0 (0.0%)	6 (15.8%)	2 (14.3%)	0 (0.0%)	3 (18.8%)
>18 months	0 (0.0%)	0 (0.0%)	0 (0.0%)	0 (0.0%)	0 (0.0%)	9 (23.7%)	0 (0.0%)	0 (0.0%)	4 (25.0%)
	Intense positive								
No breastf.	0 (0.0%)	0 (0.0%)	0 (0.0%)	0 (0.0%)	0 (0.0%)	0 (0.0%)	0 (0.0%)	0 (0.0%)	2 (50.0%)
3 months	0 (0.0%)	0 (0.0%)	0 (0.0%)	0 (0.0%)	0 (0.0%)	2 (50.0%)	0 (0.0%)	0 (0.0%)	0 (0.0%)
6 months	0 (0.0%)	0 (0.0%)	0 (0.0%)	0 (0.0%)	0 (0.0%)	0 (0.0%)	0 (0.0%)	0 (0.0%)	0 (0.0%)
12 months	0 (0.0%)	0 (0.0%)	0 (0.0%)	0 (0.0%)	0 (0.0%)	2 (50.0%)	0 (0.0%)	0 (0.0%)	1 (25.0%)
18 months	0 (0.0%)	0 (0.0%)	0 (0.0%)	0 (0.0%)	0 (0.0%)	0 (0.0%)	0 (0.0%)	0 (0.0%)	0 (0.0%)
>18 months	0 (0.0%)	0 (0.0%)	0 (0.0%)	0 (0.0%)	0 (0.0%)	0 (0.0%)	0 (0.0%)	0 (0.0%)	1 (25.0%)
*p* *	0.494	0.272	**0.019**	0.219	0.071	**0.002**	**0.002**	**0.013**	0.087

* Fisher’s Exact Test.

In Table 2, the data detail the distribution of patients in relation to different eating habits and the intensity of the identified microorganisms. Below is a breakdown of the significant findings associated with specific eating habits and the presence of certain microorganisms:

- Feeding at Fixed Hours: Statistically significant results were observed for *P. intermedia* (*p* = 0.018), *P. micros* (*p* = 0.027), and *F. nucleatum* (*p* = 0.031).
  - *P. intermedia* and *F. nucleatum*: Feeding at fixed hours was significantly more frequently associated with the presence of these microorganisms.
  - *P. micros*: The lack of feeding at fixed hours was significantly more frequently associated with results indicating the presence of this microorganism in the collected samples.
- Frequent Consumption of Sweets: Statistically significant results were only observed for *Capnocytophaga* spp. (*p* = 0.007). This microorganism was found in children with a frequent consumption of sweets.
- Proper Hydration: Statistically significant results were obtained for *T. forsythia* (*p* = 0.031), *P. micros* (*p* = 0.005), and *Capnocytophaga* spp. (*p* = 0.008).
  - *T. forsythia*, *P. micros*, and *Capnocytophaga* spp.: The presence of these microorganisms was noted in children who did not maintain age-appropriate hydration.

**Table 2:** Distribution according to various eating habits and identified microorganisms.

	*P. intermedia*	*P. gingivalis*	*T. forsythia*	*T. denticola*	*P. micros*	*F. nucleatum*	*C. rectus*	*E. corrodens*	*Capnocytophaga* spp.
Fixed hours	Negative								
No	19 (33.9%)	23 (41.1%)	13 (29.5%)	19 (36.5%)	21 (39.6%)	0 (0.0%)	8 (40.0%)	16 (36.4%)	8 (50.0%)
Yes	37 (66.1%)	33 (58.9%)	31 (70.5%)	33 (63.5%)	32 (60.45)	0 (0.0%)	12 (60.0%)	28 (63.6%)	8 (50.0%)
	Weak positive								
No	0 (0.0%)	0 (0.0%)	4 (50.0%)	4 (50.0%)	0 (0.0%)	11 (47.8%)	9 (34.6%)	7 (43.8%)	6 (25.0%)
Yes	0 (0.0%)	2 (100%)	4 (50.0%)	4 (50.0%)	5 (100%)	7 (52.2%)	17 (65.4%)	9 (56.3%)	18 (75.0%)
	Positive								
No	4 (100%)	0 (0.0%)	6 (75.0%)	0 (0.0%)	2 (100%)	12 (52.5%)	6 (42.9%)	0 (0.0%)	3 (37.5%)
Yes	0 (0.0%)	2 (100%)	2 (25.0%)	0 (0.0%)	0 (0.0%)	26 (47.5%)	8 (57.1%)	0 (0.0%)	10 (62.5%)
	Intense positive								
No	0 (0.0%)	0 (0.0%)	0 (0.0%)	0 (0.0%)	0 (0.0%)	0 (0.0%)	0 (0.0%)	0 (0.0%)	3 (75.0%)
Yes	0 (0.0%)	0 (0.0%)	0 (0.0%)	0 (0.0%)	0 (0.0%)	4 (100%)	0 (0.0%)	0 (0.0%)	1 (25.0%)
*p* *	**0.018**	0.403	0.057	0.468	**0.027**	**0.031**	0.839	0.765	0.165
Sweets	Negative								
No	23 (41.1%)	23 (41.1%)	17 (38.6%)	19 (36.5%)	21 (39.6%)	0 (0.0%)	8 (40.0%)	20 (45.5%)	12 (75.0%)
Yes	33 (58.9%)	33 (58.9%)	27 (61.4%)	33 (63.5%)	32 (60.4%)	0 (0.0%)	12 (60.0%)	24 (54.5%)	4 (25.0%)
	Weak positive								
No	0 (0.0%)	0 (0.0%)	2 (25.0%)	6 (75.0%)	4 (80.0%)	7 (38.9%)	12 (46.2%)	5 (31.3%)	7 (29.2%)
Yes	0 (0.0%)	2 (100%)	6 (75.0%)	2 (25.0%)	1 (20.0%)	11 (61.1%)	14 (53.8%)	11 (68.7%)	17 (70.8%)
	Positive								
No	2 (50.0%)	2 (100%)	6 (75.0%)	0 (0.0%)	0 (0.0%)	18 (47.4%)	5 (35.7%)	0 (0.0%)	6 (37.5%)
Yes	2 (50.0%)	0 (0.0%)	2 (25.0%)	0 (0.0%)	2 (100%)	20 (52.6%)	9 (64.35)	0 (0.0%)	10 (62.5%)
	Intense positive								
No	0 (0.0%)	0 (0.0%)	0 (0.0%)	0 (0.0%)	0 (0.0%)	0 (0.0%)	0 (0.0%)	0 (0.0%)	0 (0.0%)
Yes	0 (0.0%)	0 (0.0%)	0 (0.0%)	0 (0.0%)	0 (0.0%)	4 (100%)	0 (0.0%)	0 (0.0%)	4 (100%)
*p* *	1.000	0.148	0.107	0.057	0.111	0.208	0.842	0.386	**0.007**
Hydration	Negative								
No	10 (17.9%)	10 (17.9%)	6 (13.6%)	8 (15.4%)	6 (11.3%)	0 (0.0%)	4 (20.0%)	8 (18.2%)	4 (25.0%)
Yes	46 (82.1%)	46 (82.1%)	38 (86.4%)	44 (84.6%)	47 (88.7%)	0 (0.0%)	16 (80.0%)	36 (81.8%)	12 (75.0%)
	Weak positive								
No	0 (0.0%)	0 (0.0%)	4 (50.0%)	2 (25.0%)	2 (40.0%)	5 (27.8%)	3 (11.5%)	2 (12.5%)	1 (4.2%)
Yes	0 (0.0%)	2 (100%)	4 (50.0%)	6 (75.0%)	3 (60.0%)	13 (72.2%)	23 (88.5%)	14 (87.5%)	23 (95.8%)
	Positive								
No	0 (0.0%)	0 (0.0%)	0 (0.0%)	0 (0.0%)	2 (100%)	5 (13.2%)	3 (21.4%)	0 (0.0%)	2 (12.5%)
Yes	4 (100%)	2 (100%)	8 (100%)	0 (0.0%)	0 (0.0%)	33 (86.8%)	11 (78.6%)	0 (0.0%)	14 (87.5%)
	Intense positive								
No	0 (0.0%)	0 (0.0%)	0 (0.0%)	0 (0.0%)	0 (0.0%)	0 (0.0%)	0 (0.0%)	0 (0.0%)	3 (75.0%)
Yes	0 (0.0%)	0 (0.0%)	0 (0.0%)	0 (0.0%)	0 (0.0%)	4 (100%)	0 (0.0%)	0 (0.0%)	1 (25.0%)
*p* *	1.000	1.000	**0.031**	1.000	**0.005**	0.349	0.740	0.715	**0.008**

* Fisher’s Exact Test.

When considering the number of daily meals and its relation to the presence and intensity of different microorganisms, statistically significant outcomes were exclusively found for *Capnocytophaga* spp. A frequency of four or five meals per day was more frequently associated with results indicating its presence in the collected samples. However, for all other microorganisms, the disparities between the groups were not statistically significant according to Fisher’s test (Table 3).

Concerning the frequency of daily consumption of sweets, statistically significant associations were observed for *T. denticola* and *Capnocytophaga* spp. (as outlined in Table 3).

## 4. Discussion

Periodontal disease is influenced by a multitude of factors, including smoking, inadequate oral hygiene, hormonal fluctuations, diabetes, medication use, and stress [26]. The role of the diet in preserving the symbiosis between the oral microbiome and periodontal health is pivotal, significantly impacting periodontal conditions [12]. Notably, a sugar-rich diet escalates the risk of developing periodontal disease in both teenagers [13,27] and children [28]. An elevated sugar intake perpetuates a prolonged hyperglycemic state, escalating systemic inflammation and the susceptibility to periodontal disease [29]. Moreover, sugar consumption can alter the diversity of the oral microbiome and contribute to the development of periodontal pathogens [29]. It is widely observed that children and adolescents globally surpass current recommendations in sugar consumption [30]. Dietary preferences among the younger population vary due to diverse influences, such as familial, maternal, and paternal impacts, communal eating patterns, education, socioeconomic status, early feeding practices, and media exposure [31]. Family dynamics notably shape eating habits, while parental attitudes and behaviors toward food significantly influence a child’s dietary choices [32,33]. Higher levels of education and socio-economic status often correlate with an improved access to nutritious food and the adoption of healthier dietary habits [34]. Advertising and media wield a substantial influence over the food preferences and choices of children [35]. Notably, periodontal pathogens can be identified in newborns, children, and adolescents [36], underscoring the importance of prompt interventions aimed at mitigating periodontal pathogens and preserving a balanced oral microbiome. Regulating dietary patterns and sugar consumption and ensuring proper hydration prove to be instrumental in achieving this goal.

In this study, the micro-IDent test kit (Hain Lifescience, GmbH, Nehren, Germany) was employed to identify 11 periodontal pathogens. The choice of this test was influenced by its user-friendly sampling method and its availability in Romania. Additionally, the test is distinguished by its high specificity [10]. Previous research has successfully implemented this test to identify periodontal pathogens in various demographics, including children, adolescents [37,38], pregnant women [24], and diverse adult populations [39,40]. The 11 periodontal pathogens identified through this test have been associated with the onset of periodontal disease. Notably, *A. actinomycetemcomitans*, *P. gingivalis*, *T. forsythia*, *T. denticola*, and *F. nucleatum* pose a high risk and can induce severe forms of periodontal disease [41]. Other microorganisms, such as *P. intermedia*, *P. micros*, *E. corrodens, C. rectus*, *E. nodatum*, and *Capnocytophaga* spp., are also implicated in the occurrence and progression of periodontal disease, detectable through the utilized kit [42,43,44,45,46,47].

The investigation focused on the diverse eating habits of children. Although a majority of children adhere to scheduled mealtimes, more than a third do not follow fixed eating hours. Regular meal timings contribute to synchronizing the peripheral circadian rhythm [48]. Deviating from scheduled meals heightens the risk of obesity, metabolic disorders, and cardiovascular issues and leads to alterations in the intestinal microbiome [49]. Recognizing the impact of scheduled eating on the intestinal microbiome [49], it was hypothesized to have a similar influence on the oral microbiome. Statistically significant associations were observed in this study for *P. intermedia*, *P. micros*, and *F. nucleatum*, contrary to the authors’ expectations. Surprisingly, positive results for *P. intermedia* and *F. nucleatum* were more frequently linked to scheduled mealtimes. However, these results might also be influenced by potential misreporting by parents. There is a dearth of research investigating the role of scheduled eating on the presence of periodontal pathogens. Regarding the frequency of meals per day, over half of the children investigated consume four meals daily, while a quarter have more than five meals. Similar findings were reported by Galczak-Kondraciuk et al. (2018) regarding the number of daily meals [50]. Interestingly, *Capnocytophaga* spp. showed more frequent positive outcomes in children who consumed five meals a day. Although no comparative studies were found, this finding might be attributed to a predilection for an unbalanced diet detrimental to oral health, as this microorganism diminishes with an optimized oral health regimen [51].

Sugar significantly impacts oral health and intensifies periodontal pathogens, to the extent that the consumption of sweetened beverages can be regarded as a risk factor in the development of periodontal disease [52]. In this sample of children, the majority consumed sweets once a day, while over a third indulged in sweets 2–3 times daily. Similar levels of sugar consumption have been noted in other populations, reflecting children’s well-established preference for sugar intake [30,53]. Notably, in this sample, the presence of *Capnocytophaga* spp. showed a higher frequency when sweets were consumed 2 or 3 times a day.

The study also explored the total duration of breastfeeding among children, recognizing the known significance of breastfeeding in the early months for maintaining a stable and diverse oral microbiome [54]. Notably, the absence of breastfeeding was more frequently associated with the presence of pathogens such as *T. forsythia*, *C. rectus*, and *E. corrodens*. These findings are consistent with other research emphasizing the importance of breastfeeding in shaping a healthy oral microbiome [55].

This study offers a comprehensive examination of the influence of diet and various eating habits on the prevalence of 11 periodontal pathogens. It stands as an original investigation in its domain, characterized by a unique exploration of factors influencing periodontal pathogens in children—a facet rarely addressed in the existing literature. Notably, our results could not be directly compared with other studies due to the scarcity of similar research in this specific demographic. The distinctiveness of our study lies in its novel approach, as existing comparative data were lacking, underscoring the pioneering nature of our research in shedding light on the influence of dietary habits on periodontal pathogens in this particular sample. While serving as a foundational point for future research, it possesses certain limitations. The study’s sample size of children was relatively small, and expanding the cohort could potentially reveal more statistically significant results. Another limitation might be associated with the accuracy of parental responses, as some answers might not fully represent the children’s actual eating habits. Additionally, this study focused solely on analyzing the 11 bacteria with periodontal pathogenic potential and did not include an analysis of other oral cavity bacteria.

We acknowledge additional inherent limitations in the design of our study. Notably, our research was conducted as a cross-sectional study, which restricts making definitive causal inferences. Additionally, we recognize the presence of uncontrolled confounding variables in our study. Although we endeavored to minimize their impact, these factors might have influenced the outcomes. The potential influence of chance in our observed results is a consideration in scientific investigations. To address this concern, we employed statistical tests and conscientiously reported *p*-values and effect sizes in our findings.

Moreover, the questionnaire completed by the parents lacked a prior validation process, representing another limitation. It is important to note that our study did not utilize a convenience sample.

## 5. Conclusions

In this sample, the collected subgingival samples revealed a notable influence of eating habits and diet on the presence and intensity of specific periodontal pathogens. Notably, the absence of breastfeeding showed a higher association with the presence of *T. forsythia* and *C. rectus* in the collected samples, while inadequate age-appropriate hydration was more frequently linked to the presence of *T. forsythia* and *P. micros*. Moreover, the frequent consumption of sweets and the habit of consuming four meals a day correlated with the presence of *Capnocytophaga* spp. Considering these findings, the development of nutritional education programs aimed at parents and the implementation of an oral-health-friendly diet could prove instrumental in controlling the identified microorganisms and preventing periodontal disease.

## Figures and Tables

**Table 3:** Distribution according to the number of meals and sweets/day and identified microorganisms.

	*P. intermedia*	*P. gingivalis*	*T. forsythia*	*T. denticola*	*P. micros*	*F. nucleatum*	*C. rectus*	*E. corrodens*	*Capnocytophaga* spp.
Meals/day	Negative								
4	30 (53.6%)	30 (53.6%)	24 (54.5%)	28 (53.8%)	29 (54.7%)	9 (50.0%)	12 (60.0%)	25 (56.8%)	6 (37.5%)
5	13 (23.2%)	13 (23.2%)	13 (29.5%)	13 (25.0%)	11 (20.8%)	3 (16.7%)	2 (10.0%)	9 (20.5%)	2 (12.5%)
>5	13 (23.2%)	13 (23.2%)	7 (16.0%)	11 (21.2%)	13 (24.5%)	6 (33.3%)	6 (30.0%)	10 (22.7%)	8 (50.0%)
	Weak positive								
4	0 (0.0%)	0 (0.0%)	4 (50.0%)	4 (50.0%)	3 (60.0%)	0 (0.0%)	15 (57.7%)	7 (43.8%)	22 (91.6%)
5	0 (0.0%)	0 (0.0%)	0 (0.0%)	0 (0.0%)	2 (40.0%)	0 (0.0%)	6 (23.1%)	4 (25.0%)	1 (4.2%)
>5	0 (0.0%)	2 (100%)	4 (50.0%)	4 (50.0%)	0 (0.0%)	0 (0.0%)	5 (19.2%)	5 (31.3%)	1 (4.2%)
	Positive								
4	2 (50.0%)	2 (100%)	4 (50.0%)	0 (0.0%)	0 (0.0%)	20 (52.65)	5 (35.7%)	0 (0.0%)	2 (12.5%)
5	0 (0.0%)	0 (0.0%)	0 (0.0%)	0 (0.0%)	0 (0.0%)	9 (23.7%)	5 (35.7%)	0 (0.0%)	10 (62.5%)
>5	2 (50.0%)	0 (0.0%)	4 (50.0%)	0 (0.0%)	2 (100%)	9 (23.7%)	4 (28.6%)	0 (0.0%)	4 (25.0%)
	Intense positive								
4	0 (0.0%)	0 (0.0%)	0 (0.0%)	0 (0.0%)	0 (0.0%)	3 (75.05)	0 (0.0%)	0 (0.0%)	2 (50.0%)
5	0 (0.0%)	0 (0.0%)	0 (0.0%)	0 (0.0%)	0 (0.0%)	1 (25.0%)	0 (0.0%)	0 (0.0%)	0 (0.0%)
>5	0 (0.0%)	0 (0.0%)	0 (0.0%)	0 (0.0%)	0 (0.0%)	0 (0.0%)	0 (0.0%)	0 (0.0%)	2 (50.0%)
*p* *	0.517	0.208	0.030	0.102	0.099	0.809	0.363	0.681	**<0.001**
Sweets/day	Negative								
1	35 (62.5%)	33 (58.9%)	25 (56.8%)	29 (55.8%)	33 (62.3%)	0 (0.0%)	16 (80.0%)	29 (65.9%)	16 (100%)
2–3	20 (35.7%)	22 (39.3%)	18 (40.9%)	22 (42.3%)	19 (35.85)	0 (0.0%)	4 (20.0%)	14 (31.8%)	0 (0.0%)
>3	1 (1.8%)	1 (1.8%)	1 (2.3%)	1 (1.9%)	1 (1.9%)	0 (0.0%)	0 (0.0%)	1 (2.3%)	0 (0.0%)
	Weak positive								
1	0 (0.0%)	2 (100%)	6 (75.0%)	8 (100%)	4 (80.0%)	9 (50.0%)	15 (57.7%)	8 (50.0%)	10 (41.75)
2–3	0 (0.0%)	0 (0.0%)	2 (25.0%)	0 (0.0%)	1 (20.0%)	8 (44.4%)	11 (42.3%)	8 (50.0%)	14 (58.3%)
>3	0 (0.0%)	0 (0.0%)	0 (0.0%)	0 (0.0%)	0 (0.0%)	1 (5.6%)	0 (0.0%)	0 (0.0%)	0 (0.0%)
	Positive								
1	2 (50.0%)	2 (100%)	6 (75.0%)	0 (0.0%)	0 (0.0%)	27 (71.1%)	6 (42.9%)	0 (0.0%)	10 (62.5%)
2–3	2 (50.0%)	0 (0.0%)	2 (25.0%)	0 (0.0%)	2 (100%)	11 (28.9%)	7 (50.0%)	0 (0.0%)	6 (37.5%)
>3	0 (0.0%)	0 (0.0%)	0 (0.0%)	0 (0.0%)	0 (0.0%)	0 (0.0%)	1 (7.1%)	0 (0.0%)	0 (0.0%)
	Intense positive								
1	0 (0.0%)	0 (0.0%)	0 (0.0%)	0 (0.0%)	0 (0.0%)	1 (25.0%)	0 (0.0%)	0 (0.0%)	1 (25.0%)
2–3	0 (0.0%)	0 (0.0%)	0 (0.0%)	0 (0.0%)	0 (0.0%)	3 (75.0%)	0 (0.0%)	0 (0.0%)	2 (50.0%)
>3	0 (0.0%)	0 (0.0%)	0 (0.0%)	0 (0.0%)	0 (0.0%)	0 (0.0%)	0 (0.0%)	0 (0.0%)	1 (25.0%)
*p* *	0.649	0.439	0.642	**0.030**	0.267	0.102	0.079	0.443	**<0.001**

* Fisher’s Exact Test.

## Data Availability

The data presented in this study are available on request from the corresponding authors. The data are not publicly available due to privacy reasons.
